# Occupational exposure to organic solvents during pregnancy and childhood behavior: findings from the PELAGIE birth cohort (France, 2002–2013)

**DOI:** 10.1186/s12940-018-0406-x

**Published:** 2018-07-27

**Authors:** Nathalie Costet, Rémi Béranger, Ronan Garlantézec, Florence Rouget, Christine Monfort, Sylvaine Cordier, Fabienne Pelé, Cécile Chevrier

**Affiliations:** 1 Epidemiological Research in Environment, Reproduction and Health, Univ Rennes, Inserm, EHESP, Irset-UMR_S 1085, 9, avenue du Prof. Léon Bernard, F-35000 Rennes, France; 2 Univ Rennes, CHU Rennes, Inserm, EHESP, Irset-UMR_S 1085, Rennes, France; 3 Univ Rennes, CHU Rennes, Inserm, Irset-UMR_S 1085, CIC 1414, F-35000 Rennes, France

**Keywords:** Solvents, Prenatal exposure delayed effects, Behavior, Cohort studies

## Abstract

**Background:**

Numerous industries use organic solvents, and many workers from various occupational sectors are exposed to these known neurotoxicants, including pregnant women. Our objective is to explore whether occupational exposure of pregnant women to solvents may impair the neurodevelopment of their babies and consequently affect their behavior in childhood.

**Methods:**

Within the French birth cohort PELAGIE, parents assessed their children’s internalizing and externalizing behaviors using items from the Child Behavior Checklist and the Preschool Social Behavior Questionnaire at age 2, and the Strength and Difficulties Questionnaire at age 6. The occupational exposure to solvents of the pregnant women was self-reported prospectively at the beginning of their pregnancy (*N* = 715). We applied structural equation modeling to capture the longitudinal association of prenatal exposure to solvents with children’s behavioral traits at 2 and 6 years.

**Results:**

Increased externalizing behavior score at age 2 was associated with prenatal exposure to solvents (standardized score: 0.34 (95% CI = 0.11, 0.57) for occasional exposure and 0.26 (0.05, 0.48) for regular exposure). This association was attenuated at age 6 (0.22 (− 0.02, 0.47) for occasional exposure and 0.07 (− 0.14, 0.28) for regular exposure). No association was observed for internalizing behavior.

**Conclusions:**

Pregnant women’s occupational exposure to solvents may affect their children’s behavior in early childhood. This effect may be attenuated with aging or diluted by the effects of other postnatal predictors.

**Electronic supplementary material:**

The online version of this article (10.1186/s12940-018-0406-x) contains supplementary material, which is available to authorized users.

## Background

Organic solvents are a group of diverse chemical compounds widely used in industry due to properties convenient for extracting, dissolving, or diluting materials. Their inclusion in numerous products such as paints, degreasers, glues, inks, pharmaceuticals, pesticides, and cosmetic and cleaning products results in exposures in diverse occupational settings. In 2004, 548,000 tons of organic solvents were used in France [1]. A recent survey of a sample representative of French workers nationwide estimated that 13% of workers – 14% of men and 12% of women – were exposed to at least one solvent during the week before the visit with the occupational health physician [2]. As a nationally representative population study reported that 70% of French pregnant women work during their pregnancy [3], the issue of occupational solvent exposures in this population is particularly relevant in terms of public health.

Studies of populations exposed either occupationally or through contaminated environments provide compelling evidence of the neurotoxicity of organic solvents in humans [4, 5]. Lipid-soluble and low-molecular-weight solvents, such as toluene, xylene, benzene [6], and tetrachloroethylene [7], can easily cross the placental barrier and reach the fetus. Animal data suggest the possible neurodevelopmental toxicity of some solvents, such as toluene [8] and some low-molecular-weight glycol ethers [9]. Only three epidemiological studies have explored the impact of prenatal exposure to solvents on human neurodevelopment. Two of them suggested that children prenatally exposed to organic solvents have a higher level of behavioral problems than non-exposed children [10, 11], while another study found that the exposed children walked earlier and observed no association with behavioral problems [12]. These three studies, however, were based on a limited number of subjects (*n* = 33 to 82 children, aged 3–9 years old).

Based on data from the PELAGIE mother-child cohort’s large population-based sample with prospectively assessed exposure, we previously reported an increased level of attention deficit/hyperactivity disorders and aggression disorders among 2 year-old children whose mothers were exposed to organic solvents at work during the beginning of their pregnancy [13]. Benefiting from a longer follow-up of the children up to school age, this study examines whether the impact of prenatal occupational exposure to organic solvents on the children’s behavior persisted to age 6.

## Methods

### Population

The PELAGIE (Perturbateurs Endocriniens: Étude Longitudinale sur les Anomalies de la Grossesse, l’Infertilité et l’Enfance) mother-child cohort study has previously been described in detail [14]. Overall, 3421 pregnant women were included between 2002 and 2006 in Brittany, France, before the 19th week of gestation (median 10 weeks, interquartile range (IQR) 8–11). Mothers were asked at inclusion and when the child had turned 2 and 6 years old to complete self-administered questionnaires, focused on medical and social characteristics of the child and its family, including lifestyle, domestic habits, and occupation.

Among the 3323 women who gave birth to live-born singletons, 508 were lost to follow-up before the child turned 2. Among the 2815 remaining women, 1506 responded to the 2-year-old questionnaire, and 947 also responded to the 6-year-old questionnaire. Among these 947 women, we restricted our sample to the women who reported working at the beginning of the pregnancy (*N* = 866). We excluded families if the child was born before the 35th week of gestation (N = 8), had a chromosomal anomaly (*N* = 1), had severe head trauma during childhood (N = 1), or if a parent or sibling died during childhood (father: *N* = 2; sibling: *N* = 3), as well as children with missing behavior scores at age 2 and/or age 6 (*N* = 58). The eligible sample included 851 children (see Flowchart in Additional file 1: Figure S1).

All mothers provided written informed consent, and the appropriate French ethics committees approved the study procedures.

### Assessment of child behavior

At the 2-year-old follow-up, parents assessed their children’s behavior using items derived from the Child Behavior Checklist (CBCL) [15, 16] and the Preschool Social Behavior Questionnaire (PSBQ) [17]. Items were 3-point Likert scales ranging from 0 to 2 points (0 = never, 1 = sometime, 2 = often), summed into four different subscales: a score for the attention deficit/hyperactivity subscale was computed from six items, and scores for the aggression, opposition, and emotionality subscales were derived from 3 items each. The detailed list of items is presented in Additional file 1: Table S1, further details about construction of the subscales are provided elsewhere [13].

The year the children turned 6, parents assessed their behavior using four subscales of the French version of the Strengths and Difficulties Questionnaire (SDQ) [18, 19]: emotional symptoms, peer relationship problems, conduct problems, and hyperactivity-inattention. These subscales were derived from 20 items scored from 0 (not true) to 2 (certainly true). When children had two missing SDQ items or more within a subscale (*N* = 3), no score was calculated. In 40 children with one missing item, the corresponding subscale score was extrapolated from the remaining available items. The sample included 793 children with available behavior subscales.

For all subscales at age 2 and 6, higher scores indicate more potential problems.

### Exposure assessment

At inclusion (≤ 19 weeks of gestation), women were asked whether in their current job, they were using, producing, or exposed to one of 11 groups of products known to contain organic solvents (paints, strippers, varnishes, dyes, inks, glues, gasoline, grease remover, detergents and cleaning agents, textile treatment agents, and cosmetics) according to a 3-level scale (never, occasionally, regularly). Women reporting regular exposure to at least one group of products were considered “regularly exposed”; women reporting occasional exposure to at least one group of products were considered “occasionally exposed”; and finally, women were classified “not exposed” if they reported no exposure to any of these products [14]. The exposure status was available for 95.5% of the working women. We then excluded the 42 women who declared at the 2-year follow-up that they had changed job position in the late pregnancy. The final sample for analysis included 715 mother-child pairs.

### Statistical analysis

We addressed the relations between the multidimensional and longitudinal child’s behavior data within the structural equation modeling (SEM) framework.

**Fig. 1:**
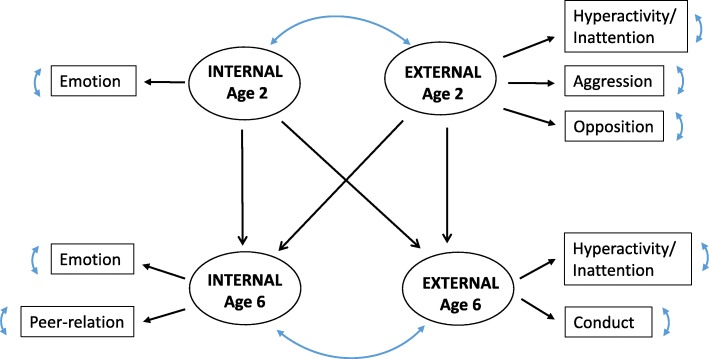
Structural Equation Modeling of behavior traits at ages 2 and 6, raw model, PELAGIE cohort, France, 2002–2013. Abbreviations: Latent traits: Internal, internalizing behavior; External, externalizing behavior. Observed variables are scores from the Child Behavior Checklist (CBCL) and the Preschool Social Behavior Questionnaire (PSBQ) at age 2 and from the Strengths and Difficulties Questionnaire (SDQ) at age 6. At age 2: Emotion, “Emotional symptoms” (CBCL); Hyperactivity/Inattention, “Attention deficit/hyperactivity” (CBCL); "Aggression" (CBCL); "Opposition" (CBCL). At age 6: Emotion, “Emotional symptoms”, Peer-relation, “Peer-relationship problems”; “Hyperactivity/Inattention”; Conduct, “Conduct problems”. Arrows relating latent traits and observed variables represent standardized factor loadings. Double-headed curvilinear arrows represent covariances between latent traits at each age or residual variances of observed variables. Arrows between latent traits of different ages represent standardized regression coefficients. Each latent trait at age 6 was regressed on the two latent traits at age 2

To deal with the multidimensional data, we defined latent variables representing internalizing, and externalizing behaviors at age 6, as suggested by Goodman et al. [20] for the SDQ, and we applied the same rationale at age 2 (Fig. 1). The internalizing behavior trait was thus defined at age 2 by the emotional symptoms subscale, at age 6 by the emotional symptoms and peer relationship problems subscales. The externalizing behavior trait was defined at age 2 by the attention deficit/hyperactivity, aggression and opposition subscales, at age 6 by the hyperactivity/inattention and conduct problems subscales. Covariance between the two latent traits at each age was estimated.

The longitudinal approach was considered by regressing each latent trait at age 6 linearly on the two latent traits at age 2, and by decomposing the associations between exposure and behavior latent traits at age 6 (total associations) into direct and indirect pathways. The indirect pathway represents the associations at age 6 that are determined by the associations between exposure and behavior traits at age 2 and the correlations between behavior traits at age 2 and 6. The direct pathway represents the marginal association at age 6, after adjusting for the associations at age 2.

First, a crude structural model representing the relations between latent behavior traits at ages 2 and 6 was fitted (Fig. 1). Each latent trait at age 6 was regressed linearly on all the two latent traits at age 2. All models’ parameters were estimated with a weighted least squares procedure (WLSM), which provides robust estimators and standard errors when the normality assumption is violated for observed variables [21]. Factor loadings and latent traits were standardized for easier interpretation. Parameters were considered significant if their 95% confidence intervals (95% CIs) did not include 0. A Satorra–Bentler scaled Chi-square statistic with *P* > 0.05, a RMSEA< 0.06, a CFI > 0.9, a GFI > 0.9 and a SRMR< 0.05 were considered to indicate good fit of the model [22].

In a second phase, we included the prenatal solvent exposure variable as a categorical variable. Both latent behavior traits at age 2 and age 6 were linearly regressed on the exposure variable. The association between exposure and each latent behavioral trait is interpreted as the mean change in the latent trait (expressed in number of standard deviations), expected for exposed compared to unexposed children.

All regression parameters were a priori adjusted for maternal age (continuous); maternal education (≤12 years, > 12 years); parity (0, ≥1); child’s sex; maternal tobacco consumption at the beginning of pregnancy (no, < 10 cig/day, ≥10 cig/day); breastfeeding (none, ≤16, > 16 weeks); mother-child interaction at age 2. This variable was based on five items collected at the 2-year follow-up on the activities shared with the child (e.g., singing, playing, and reading) (see Additional file 1: Table S2; see also Pelé et al. [13]). Missing values for covariates (< 3.6%) were imputed by the mode for categorical variables, and by the median for continuous variables.

Finally, we stratified our analyses by sex to explore possible differential pathways and associations in boys and girls. We tested the measurement invariance of the latent behavioral traits between boys and girls by comparing a restricted model constrained to estimate equal factor loadings in both sexes and the unrestricted model. The chi-square change (and associated *p*-value) was used to state whether measurement models were equivalent. SEM analyses were performed with the lavaan package, R software V3.3.0 [23].

## Results

Table 1 presents the demographic characteristics and the lifestyle factors of the 715 mother-child pairs. At the beginning of the pregnancy, most mothers were non-smokers (77%) and had completed a post-secondary degree (87%). Their mean age at delivery was 30.8 years, 42% had no previous delivery, 16% were overweight, and 21% had breastfed the child for more than 6 weeks. Fifty-three percent of children were boys. Overall, about half the pregnant women reported occupational exposure to organic solvents, either occasionally (19%) or regularly (31%) (Table 1). These exposures were mainly cleaning products and detergent (63% of the occasionally exposed women and 79% of the regularly exposed), glues, mastics, resins and adhesives (38 and 34%, respectively), inks and dyes (17 and 21%, respectively), or diluent and grease remover (10 and 24%, respectively) (see Additional file 1: Table S3). Among women classified as occasionally (respectively regularly) exposed to solvents, 63% (69%) were exposed occasionally (regularly) to one group of products, 21% (20%) to two groups and 16% (11%) to 3 groups of solvents or more. Among women classified as regularly exposed, some of them cumulated occasional exposure to other groups of solvents, so that 40% of them were exposed regularly and/or occasionally to one group of products, 27% to two groups, 33% to 3 groups of solvents or more (data not shown).

**Table 1:** Characteristics of the Study Population (PELAGIE Cohort, France, 2002–2013, *N* = 715)

	N	Mean ± SD or n (%)
Maternal age (years)	715	30.8 ± 4.0
Maternal education level	714	
< 12 years		84 (11.7)
12 years		114 (16.0)
> 12 years		516 (71.3)
Maternal prepregnancy body mass index	715	
≤ 25 kg/m^2^		604 (84.5)
> 25 kg/m^2^		111 (15.5)
Maternal tobacco consumption (early pregnancy)	710	
None		547 (77.0)
< 10 cig/day		118 (16.6)
≥ 10 cig/day		45 (6.3)
Maternal occupational exposure to solvents	715	
No		356 (49.8)
Occasional		139 (19.4)
Regular		220 (30.8)
Parity	715	
Nulliparous		299 (41.8)
Parous		416 (58.2)
Sex	715	
Boys		379 (53.0)
Girls		336 (47.0)
Breastfeeding	715	
None		232 (32.4)
< 6 weeks		331 (45.3)
≥ 6 weeks		152 (21.3)
Mother-child interaction at age 2^a^	695	19.8 ± 3.5

^a^Score varying between 5 and 25 (higher is better). For details, see Additional file 1: Table S2

Compared to the sample at inclusion in the cohort PELAGIE, women included in the present study tended to be older (+ 0,8 year on average), to have higher educational level (87% vs 81%), to be more often non-smokers during pregnancy (77% vs 72%), and slightly less overweight (16% vs 17%) and nulliparous (42% vs 44%). However, the distribution of prenatal exposure to solvents and behavioral scores remained very similar through the different follow-ups of the children of the cohort (see Additional file 1: Table S5).

Median behavior subscale scores at the ages of 2 and 6 are reported in Table 2, together with their loadings (λ) on their corresponding latent behavioral trait (crude model). Because the internalizing disorders latent trait was related to only one observed variable, its loading at age 2 was set to 1, by definition. At age 2, the externalizing behavior trait was highly related to the attention deficit/hyperactivity score (λ = 0.70), then to the opposition score (λ = 0.63) and moderately to the aggression score (λ = 0.44). At age 6, the internalizing behavior trait was related to both emotional (λ = 0.63) and peer-relational problems (λ = 0.52) and the externalizing behavior trait was highly related to conduct problems (λ = 0.71) and then to the hyperactivity score (λ = 0.61).

**Table 2:** Description of the study population

	Area 1 Grabouw n (%)	Area 2 Piketberg n (%)	Area 3 DeDoorns n (%)	Total n (%)
No. of participants	325 (32.5)	303 (30.3)	373 (37.2)	1001 (100)
Age categories				
9-11 years	194 (59.7)	223 (73.6)	175 (46.9)	592 (59.1)
12-14 years	116 (35.7)	79 (26.1)	161 (43.2)	356 (35.6)
15-16 years	15 (4.6)	1 (0.3)	37 (9.9)	53 (5.3)
Gender				
Female	170 (52.3)	159 (52.5)	199 (53.4)	528 (52.7)
Male	155 (47.7)	144 (47.5)	174 (46.6)	473 (47.2)
No. of schools	3 (42.8)	2 (28.6)	2 (28.6)	7 (100)
Grade categories				
2^nd^-3^rd^	37 (11.4)	77 (25.4)	49 (13.1)	163 (16.3)
4^th^-6^th^	235 (72.3)	210 (69.3)	222 (59.5)	667 (66.6)
7^th^-9^th^	53 (16.3)	16 (5.3)	102 (27.3)	171 (17.1)
Current Farm resident	202 (62.2)	121 (39.9)	142 (38.1)	465 (46.4)
Occupation				
Family member works on a farm	199 (61.2)	180 (59.4)	281 (75.3)	660 (65.9)
Pesticide activities				
Seen pesticide spraying activities in nearby field	278 (34.6)	233 (76.9)	291 (78)	802 (80.1)
Helped with cleaning farm equipment	63 (85.5)	63 (20.8)	97 (26)	223 (23.2)
Assisted with pesticide storage in the past 7 days	65 (20)	49 (16.2)	92 (24.7)	206 (20.5)
Social Media use				
Use a mobile phone	187 (57.5)	63 (20.8)	68 (18.2)	318 (31.7)
Use a smart phone	176 (54.2)	48 (15.8)	60 (16.1)	284 (28.4)
Connect to the internet to watch videos	112 (34.5)	16 (5.9)	17 (4.6)	145 (14.5)
Connect to the internet to play online games	101 (31.1)	6 (2)	17 (4.6)	124 (12.4)
Connect to the internet to listen to music	111 (34.2)	13 (4.3)	21 (5.6)	145 (14.5)

Each behavior latent trait at age 6 was significantly associated with the same latent trait at age 2 (standardized regression coefficients: 0.31 (95% CI = 0.19, 0.42) for internalizing behavior; and 0.66 (95% CI = 0.56, 0.77) for externalizing behavior) (see Additional file 1: Table S6). We also found a statistically significant relation between the externalizing behavior trait at age 2 and the internalizing behavior trait at age 6 (0.21, 95% CI = 0.07, 0.34). The fit statistics indicated that the model fit the data satisfactorily (RMSEA = 0.043, GFI = 0.998, CFI = 0.978, CFI = 0.978). The adjustment of the model for covariates and exposure variable did not modify these structural relations: after adjustment, we found similar factor loadings (see Additional file 1: Table S7) and associations between behavioral latent traits at ages 2 and 6 (see Additional file 1: Table S6).

**Fig. 2:**
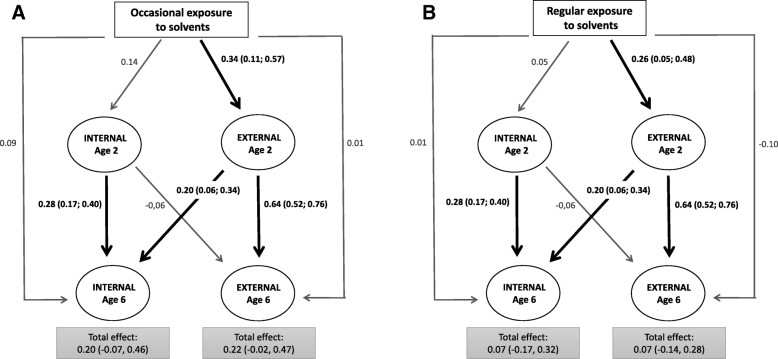
Associations of occasional (**a**) and regular (**b**) prenatal self-reported exposure to solvents with behavior traits at ages 2 and 6 (*N* = 715, PELAGIE cohort, France, 2002–2013), compared to non-exposure. Latent traits: Internal, internalizing behavior; External, externalizing behavior. All regression coefficients were adjusted for sex, education level, maternal age, breastfeeding duration, smoking during pregnancy, parity and mother-child interaction score. Results are presented in Table 3. Bold arrows indicate significant standardized regression coefficients (and confidence intervals). The association of latent traits at age 2 with prenatal exposure can be directly read on the graph arrows on the top of the graph. The direct association of latent traits at age 6 with prenatal exposure can be directly read on the broken arrows around the graphs. The indirect associations of each latent trait at age 6 can be derived by summing the associations through the two pathways pointing to it via internal and external traits at age 2. Each of these pathways generates an association with exposure. The intensity of the association is given by multiplying all the regression coefficients situated on the respective pathway. The total association, indicated in the shaded boxes under each latent trait at age 6, is obtained by summing the direct and indirect associations with exposure. Example of decomposition of the total association of occasional exposure to solvents with externalizing behavior at age 6: Direct association = 0.01; Indirect association via internalizing behavior trait at age 2 = (0.14*-0.06); via externalizing behavior trait at age 2 = (0.34*0.64). Indirect association = (0.14*-0.06) + (0.34*0.66) = 0.224. Total association = − 0.008 + 0.224 = 0.216

The associations between exposures and behavior traits through age 6 are presented in Table 3 and Fig. 2. Scores of externalizing behavior trait at age 2 were higher among children whose mothers reported occasional and regular occupational exposure to organic solvents during pregnancy (respectively, 0.34, 95% CI = 0.11, 0.57 and 0.26, 95% CI = 0.05, 0.48) than among unexposed mothers. No statistically significant association was observed between exposure and the internalizing behavior trait at age 2.

**Table 3:** Associations Between Occupational Solvent Exposure During Pregnancy and Child Behavior Traits at Ages 2 and 6 (*N* = 715, PELAGIE Cohort, France, 2002–2013)

			Association at age 2	Association at age 6	
				Total (Direct + indirect pathways)	Direct pathway
Behavior traits	Self-reported exposure	N	Stand. coeff.^a^ (95% CI)	Stand. Coeff.^a^ (95% CI)	Stand. Coeff.^a^ (95% CI)
Internalizing behavior	None	364	ref	ref	ref
	Occasional	142	0.14 (−0.05, 0.32)	0.20 (−0.07, 0.46)	0.09 (−0.17, 0.35)
	Regular	229	0.05 (0.12, 0.23)	0.07 (−0.17, 0.32)	0.01 (−0.23, 0.25)
Externalizing behavior	None	364	ref	ref	ref
	Occasional	142	0.34 (0.11, 0.57)	0.22 (−0.02, 0.47)	0.01 (−0.22, 0.25)
	Regular	229	0.26 (0.05, 0.48)	0.07 (−0.14, 0.28)	−0.10 (− 0.30, 0.11)

*Abbreviations*: 95% CI, 95% Confidence Interval; Stand. Coeff., SEM-based adjusted standardized regression coefficients

^a^SEM standardized regression coefficients, expressed in number of SD of the latent trait. All SEM regression models were adjusted for sex, education level, maternal age, breastfeeding duration, smoking during pregnancy, parity, and mother-child interaction score. Fit indices: Chi Square = 117.894, df = 63, *P* < =0.01; RMSEA = 0.035; GFI = 0.999; CFI = 0.953; SRMR = 0.027

At age 6, the associations between occasional and regular prenatal exposure and the externalizing behavior score were lower than at age 2 and statistically non-significant (respectively, 0.22, 95% CI = (− 0.02, 0.47) and 0.07 (95% CI = (− 0.14, 0.28)) compared to non-exposure. No association was observed for the internalizing behavior trait at age 6.

After adjustment for the latent behavior traits at age 2, the marginal associations (direct effect) of prenatal exposure with the latent behavior traits at age 6 were all non-significant. The fit statistics of the model were satisfactory (RMSEA = 0.035, GFI = 0.999, CFI = 0.953).

Stratified analyses according to sex showed that the SEM model fit better in girls than in boys (Additional file 1: Tables S8-S10). The measurement invariance of the latent behavioral traits between boys and girls was rejected (*P* = 0.02), meaning that latent behavior variables did not measure the behavior traits similarly (factor loadings were not strictly equal) (Additional file 1: Table S8). This is particularly true at age 6, where the peer-relationship score was predominant to define the externalized behavior in boys (factor loading = 0.78 vs 0.60 in girls) and the hyperactivity-inattention score was predominant to define the internalized behavior in girls (0.76 vs 0.58 in boys). We also observed that the association between externalizing trait at age 2 and internalizing trait at age 6 was lower and not statistically significant in girls, compared to boys (Additional file 1: Table S9). Despite these structural differences which prevented us from formally concluding about differential effect prenatal exposure, we observed that the association between prenatal exposure to solvents and the externalizing behavior trait at age 2 was stronger in girls (0.44, 95% CI: 0.11, 0.77) than in boys (0.23, 95% CI = − 0.09, 0.55) (Additional file 1: Table S10).

## Discussion

Higher scores of externalizing behavior disorders were seen among 2-year-old children whose mothers were exposed to organic solvents at work during their pregnancy. This association appeared strongly attenuated at age 6. No association was found between internalizing behavior at age 2 or age 6 and self-reported prenatal exposure to solvents.

As expected, the present study confirms our previous findings conducted among 2-year-old children in the PELAGIE cohort [13]. It additionally suggests that the possible role of occupational exposure to solvents during pregnancy on child externalizing behavior at age 2 may be transient. One hypothesis is that the effect may be reversible. More likely, possible compensatory mechanisms and/or the experience during childhood of different stimuli and stressors (all of them unmeasured) that may impact neuro-development, might have diluted the adverse effect of the prenatal exposure observed at age 2. To our knowledge, no similar study with repeated measurements of behavioral traits in childhood has been conducted previously to document that point.

Only a few previous studies were conducted and have suggested impaired behavior among children prenatally exposed to organic solvents. Laslo-Baker et al. found higher scores for both internalizing and externalizing disorders with the CBCL scale, based on 32 exposed and 32 unexposed children aged 3–9 years. They also observed higher levels of hyperactivity in exposed children based on the Conner’s Rating Scale-Revised [11]. Using the CBCL scale, Till et al. reported that children aged 3–7 years who had been prenatally exposed to organic solvents (*n* = 33) were at higher risk of both externalizing or internalizing behavioral problems, compared to unexposed children (*n* = 28) (*P* = 0.02 and *P* < 0.01, respectively) [10]. Both studies used self-reported exposure measurements standardized within a large-scale counseling program for pregnant women on diverse risks related to drugs, chemicals, radiation, and infections. Among the exposed groups, the main occupations were factory workers, laboratory technicians, graphic designers, and photography laboratory workers who had regular exposure (at least 5 h/week in Till et al. [10]) to multiple organic solvents including aromatic hydrocarbons (e.g., toluene, benzene, and xylene), alcohols (e.g., ethanol, methanol, and isopropanol) and aliphatic hydrocarbons (e.g., methane and ethane) and, especially in Till et al. [10], halogenated compounds (e.g., trichloroethylene and methyl chloride).

Conversely, using the Conners Parent Scale of Hyperactivity and the National Institute of Mental Health (NIHM) Childhood Personality Scale-Revised, Eskenazi et al. found no association between in utero exposure to organic solvents and behavior among 3.5 year-olds (*n* = 41 exposed and 41 unexposed) [12]. In this study, two independent industrial hygienists assessed exposure based on job title and industry and on the associated job description, including the material and equipment used. Occupations in the exposed group were mainly lab-workers, art-related workers, and operating room personnel, with identified exposures to aromatic hydrocarbons and halogenated solvents.

In the PELAGIE cohort, the women occasionally exposed were most frequently teachers (22%), clerical and related workers (19%), and health workers (assistant nurses, nurses, midwifes and x-ray technicians) (13%). The women regularly exposed were most frequently nurses’ aides, nurses, midwifes and x-ray technicians (29%), cleaners and helpers (13%), teachers (11.4%), and chemists and biologists (8%) (Additional file 1: Table S4). These occupations differ from the occupations represented in the similar studies above-mentioned [10–12]. Teachers mainly reported exposure to glues, mastics, adhesives (42%), inks and dyes (27%), paints and lacquers (25%). Health workers reported mainly exposure to cleaning products or detergents (82%). Clerical workers reported exposure to glues, mastics, resins and adhesives (13%) or detergents (13%) and inks (7%). These groups of workers are likely to be exposed mostly to oxygenated and chlorinated solvents that may be associated with widely different potencies in regard to developmental neurotoxicity.

There were limited differences in the product types declared by women reporting occasional or regular exposures (detergents and cleaning agents, glues, dyes or inks, and diluents or grease removers) (see Additional file 1: Table S3). This suggests some exposure similarities in terms of chemical compound classes. However, the differential occupations correspond probably to different conditions and intensities of exposure, different uses of individual protection, and exposure to different (unmeasured) mixtures of compounds.

A major limitation of our study is that the exposure assessment was based on maternal self-reports and that groups were defined according to frequency and not to intensity of exposure. Nonetheless, we previously reported positive and statistically significant associations between this maternal self-report of regular occupational exposure and biomarkers of some types of solvents known to be used occupationally (e.g., glycol ethers and halogenated solvents), which supports the reliability of this self-reported exposure measurement [24].

The fact that the behavior scores at age 2 were adapted from two scales, the CBCL and the PSBQ, and were thus not strictly validated, may also be a limitation of this study. However, satisfactory Cronbach’s alpha scores for these behavioral scores were previously estimated in our cohort, which suggests good reliability [13]. Despite the use of different behavioral tools at ages 2 and 6, internalizing and externalizing behavior traits at age 2 were positively and statistically significantly correlated with their corresponding traits at age 6. This homotypic continuity for externalizing disorders is consistent with previous studies among children of similar ages or older [25, 26]. Finally, parents’ perception of their children’s behavior might depend on the sex, as shown in a validation study of the SDQ among children aged 5–6 years [27]. This may result in the lack of measurement invariance between sexes that we observed, with latent traits being differentially measured in boys and girls. This prevented us from formally concluding about the possible heterogeneous association between exposure and externalizing behavior for girls and boys.

In our analyses, we could not consider other possible sources of maternal exposure to solvents during pregnancy and postnatal exposure of the children at home or outside, except the use of products containing solvents for household renovation during childhood, which was not correlated with prenatal occupational exposure in our data.

Our study also presents strengths. The PELAGIE cohort, specifically designed to explore the possible role of prenatal exposure to various chemicals on the childhood development, assessed exposure prospectively and makes it possible to consider trajectories of different behavioral traits between ages 2 and 6 questioning the persistence of the associations observed in early childhood in previous work of the cohort. This study was conducted on a sub-sample (*n* = 715 participants to the 2 and 6-year-old follow-ups) of our previous work (*n* = 1278 participants to the 2-year-old follow-up). However, behavioral scores and maternal exposure distribution at age 2 were similar between participants and non-participants at the 6-year-old follow-up (see Additional file 1: Table S11); possible selection bias is thus likely to be very minimal. Our methodological approach makes it possible to analyze different age-specific multi-item behavioral scales without aggregating them in predetermined single scores at each age, and in this sense, we better handle possible measurement errors of the behavior traits. This approach has also allowed us to avoid the multiple testing that occurs when separate regression models are fitted repeatedly for different outcomes. Finally, this is a large sample size (n = 715) compared to the few previous studies conducted on this topic (*n* < 100).

## Conclusions

We confirmed the observation of higher scores of externalizing behavior at age 2 in children whose mothers have been occupationally exposed to organic solvents at the beginning of their pregnancy. Among our general-population-based cohort, this association seemed attenuated at age 6, suggesting that compensatory mechanisms may occur, and/or that many other unmeasured stimuli and stressors during childhood affecting behavior may dilute the effect of prenatal exposure to solvents. These results should encourage further studies to identify the compounds or mixtures of compounds associated for these results and increased awareness in occupational settings for preventive chemical protection of pregnant women.

## Additional file

Additional file 1: **Figure S1.** Flow chart of sample selection (PELAGIE Cohort, France, 2002–2013). **Table S1.** Items used to assess the child behavior at age 2 in the PELAGIE cohort study, France, 2002–2013. **Table S2.** Items used to assess the mother-child interaction at age 2 in the PELAGIE cohort study, France, 2002–2013. **Table S3.** Groups of products related to occupational exposure to solvents (*n* = 715) in the PELAGIE Cohort study, France, 2002–2013. **Table S4.** Distribution of maternal occupations during pregnancy in the PELAGIE Cohort, France, 2002–2013. **Table S5.** Characteristics of samples across the PELAGIE Cohort follow-ups (France, 2002–2013). **Table S6**. Associations between behavior latent traits at 2 and 6 years (crude and adjusted models), PELAGIE Cohort, France, 2002–2013, *N* = 715. **Table S7**. Structural Equation Modeling of behavior at ages 2 and 6 in the PELAGIE Cohort, France, 2002–2013 – Factor loadings for the latent traits at ages 2 and 6 (crude and adjusted model, n = 715). **Table S8**. Structural Equation Modeling of behavior at ages 2 and 6 in the PELAGIE Cohort. France. 2002–2013 – Factor loadings for the latent traits at ages 2 and 6 (crude model) in boys (*N* = 379) and girls (*N* = 336). **Table S9**. Associations between behavior latent traits at age 6 and age 2 in boys (N = 379) and girls (N = 336) (crude model), PELAGIE Cohort, France, 2002–2013. **Table S10**. Sex-Stratified Total Associations Between Occupational Solvent Exposure During Pregnancy and Child Behavior at Age 2 and Age 6 (N = 379 Boys and N = 336 Girls, PELAGIE cohort, France, 2002–2013). **Table S11**. Comparison of children participating at the 2 year and 6 year follow-ups with those participating only at the 2 year follow-up (PELAGIE Cohort, France, 2002–2013). (DOCX 131 kb)

## Abbreviations

95% CI: 95% confidence interval
CBCL: Child Behavior Checklist
GFI: Goodness of fit index
IQR: Interquartile range
NIHM: National Institute of Mental Health
PSBQ: Preschool Social Behavior Questionnaire
RMSEA: Root Mean Square Error Approxiamtion
SDQ: Strengths and Difficulties Questionnaire
SEM: Structural Equation Modelling
SRMR: Standardized root-mean-square residual
WLSM: Weighted least squares-mean
