# Correction to: Occupational exposure to organic solvents during pregnancy and childhood behavior: findings from the PELAGIE birth cohort (France, 2002–2013)

**DOI:** 10.1186/s12940-018-0415-9

**Published:** 2018-09-04

**Authors:** Nathalie Costet, Rémi Béranger, Ronan Garlantézec, Florence Rouget, Christine Monfort, Sylvaine Cordier, Fabienne Pelé, Cécile Chevrier

**Affiliations:** 1 Epidemiological Research in Environment, Reproduction and Health, Univ Rennes, Inserm, EHESP, Irset-UMR_S 1085, 9, avenue du Prof. Léon Bernard, F-35000 Rennes, France; 2 Univ Rennes, CHU Rennes, Inserm, EHESP, Irset-UMR_S, 1085 Rennes, France; 3 Univ Rennes, CHU Rennes, Inserm, Irset-UMR_S 1085, CIC 1414, F-35000 Rennes, France

## Correction

Following publication of the original article [1], the author noticed that a mistake occured during the final production step and asked to replace Table 2 with the correct version.

**Table 2:** Behavior Subscales Observed at Age 2 and 6 (Observed Variables) and Loadings on Their Corresponding Latent Trait (SEM Crude Model, *N*=715, PELAGIE Cohort, France, 2002-2013)

Behavior subscales	Scale of origin ^a^	# items	Median scores (IQR)	Corresponding behavior latent trait	Factor loading
					λ ^b^ (95% CI)
At age 2					
Emotional symptoms	CBCL	3	2 (0 – 2)	Internalizing	1.00
Attention deficit/hyperactivity	CBCL, PSBQ	6	5 (3 – 7)	Externalizing	0.70 (0.63, 0.78)
Aggression	CBCL	3	1 (0 – 3)	Externalizing	0.44 (0.36, 0.53)
Opposition	CBCL	3	3 (2 – 4)	Externalizing	0.63 (0.57, 0.70)
At age 6					
Emotional symptoms	SDQ	5	2 (1 – 3)	Internalizing	0.63 (0.51, 0.74)
Peer relationship problems	SDQ	5	1 (0 – 2)	Internalizing	0.52 (0.42, 0.62)
Hyperactivity/inattention	SDQ	5	3 (1 – 4)	Externalizing	0.61 (0.53, 0.69)
Conduct problems	SDQ	5	2 (1 – 3)	Externalizing	0.71 (0.63, 0.78)

*Abbreviations: IQR,* interquartile range (25^th^ percentile – 75^th^ percentile)

^a^CBCL: Child Behavior Checklist; PSBQ: Preschool Social Behavior Questionnaire; SDQ: Strengths and Difficulties Questionnaire

^b^Standardized factor loading (95% Confidence Interval)

The correct version of the table has been included in this correction.

The original article has been corrected.
