# Supplementary material for: Occupational exposure to organic solvents during pregnancy and childhood behavior: findings from the PELAGIE birth cohort (France, 2002–2013)
Source: Environ Health. 2018 Jul 27;17:63. doi: 10.1186/s12940-018-0406-x (PMC6062867; doi:10.1186/s12940-018-0406-x)
Supplement: Supplementary file 1 — Figure S1. Flow chart of sample selection (PELAGIE Cohort, France, 2002–2013). Table S1. Items used to assess the child behavior at age 2 in the PELAGIE cohort study, France, 2002–2013. Table S2. Items used to assess the mother-child interaction at age 2 in the PELAGIE cohort study, France, 2002–2013. Table S3. Groups of products related to occupational exposure to solvents (n = 715) in the PELAGIE Cohort study, France, 2002–2013. Table S4. Distribution of maternal occupations during pregnancy in the PELAGIE Cohort, France, 2002–2013. Table S5. Characteristics of samples across the PELAGIE Cohort follow-ups (France, 2002–2013). Table S6. Associations between behavior latent traits at 2 and 6 years (crude and adjusted models), PELAGIE Cohort, France, 2002–2013, N = 715. Table S7. Structural Equation Modeling of behavior at ages 2 and 6 in the PELAGIE Cohort, France, 2002–2013 – Factor loadings for the latent traits at ages 2 and 6 (crude and adjusted model, n = 715). Table S8. Structural Equation Modeling of behavior at ages 2 and 6 in the PELAGIE Cohort. France. 2002–2013 – Factor loadings for the latent traits at ages 2 and 6 (crude model) in boys (N = 379) and girls (N = 336). Table S9. Associations between behavior latent traits at age 6 and age 2 in boys (N = 379) and girls (N = 336) (crude model), PELAGIE Cohort, France, 2002–2013. Table S10. Sex-Stratified Total Associations Between Occupational Solvent Exposure During Pregnancy and Child Behavior at Age 2 and Age 6 (N = 379 Boys and N = 336 Girls, PELAGIE cohort, France, 2002–2013). Table S11. Comparison of children participating at the 2 year and 6 year follow-ups with those participating only at the 2 year follow-up (PELAGIE Cohort, France, 2002–2013). (DOCX 131 kb) [file 12940_2018_406_MOESM1_ESM.docx]

**Supplemental Material**

**Occupational exposure to organic solvents during pregnancy and childhood behavior: findings from the PELAGIE birth cohort (France, 2002-2013).**

Nathalie Costet*^1^, Rémi Béranger*^2^, Ronan Garlantézec^2^, Florence Rouget^2^, Christine Monfort^1^, Sylvaine Cordier^1^, Fabienne Pelé^2^, Cécile Chevrier^1^.

**Table of Contents**

**Figure S1.** Flow chart of sample selection (PELAGIE Cohort, France, 2002-2013)

**Table S1.** Items used to assess the child behavior at age 2 in the PELAGIE cohort study, France, 2002-2013.

**Table S2.** Items used to assess the mother-child interaction at age 2 in the PELAGIE cohort study, France, 2002-2013.

**Table S3.** Groups of products related to occupational exposure to solvents (n= 715) in the PELAGIE Cohort study, France, 2002-2013.

**Table S4.** Distribution of maternal occupations during pregnancy in the PELAGIE Cohort, France, 2002-2013.

**Table S5.** Characteristics of samples across the PELAGIE Cohort follow-ups (France, 2002-2013).

**Table S6**. Associations between behavior latent traits at 2 and 6 years (crude and adjusted models), PELAGIE Cohort, France, 2002-2013, N=715.

**Table S7**. Structural Equation Modeling of behavior at ages 2 and 6 in the PELAGIE Cohort, France, 2002-2013 – Factor loadings for the latent traits at ages 2 and 6 (crude and adjusted model, n=715).

**Table S8**. Structural Equation Modeling of behavior at ages 2 and 6 in the PELAGIE Cohort. France. 2002-2013 – Factor loadings for the latent traits at ages 2 and 6 (crude model) in boys (N=379) and girls (N=336).

**Table S9**. Associations between behavior latent traits at age 6 and age 2 in boys (N=379) and girls (N=336) (crude model), PELAGIE Cohort, France, 2002-2013.

**Table S10**. Sex-Stratified Total Associations Between Occupational Solvent Exposure During Pregnancy and Child Behavior at Age 2 and Age 6 (N=379 Boys and N=336 Girls, PELAGIE cohort, France, 2002-2013).

**Table S11**. Comparison of children participating at the 2 year and 6 year follow-ups with those participating only at the 2 year follow-up (PELAGIE Cohort, France, 2002-2013).

**Figure S1. Flow chart of sample selection (PELAGIE Cohort, France, 2002-2013)**

**
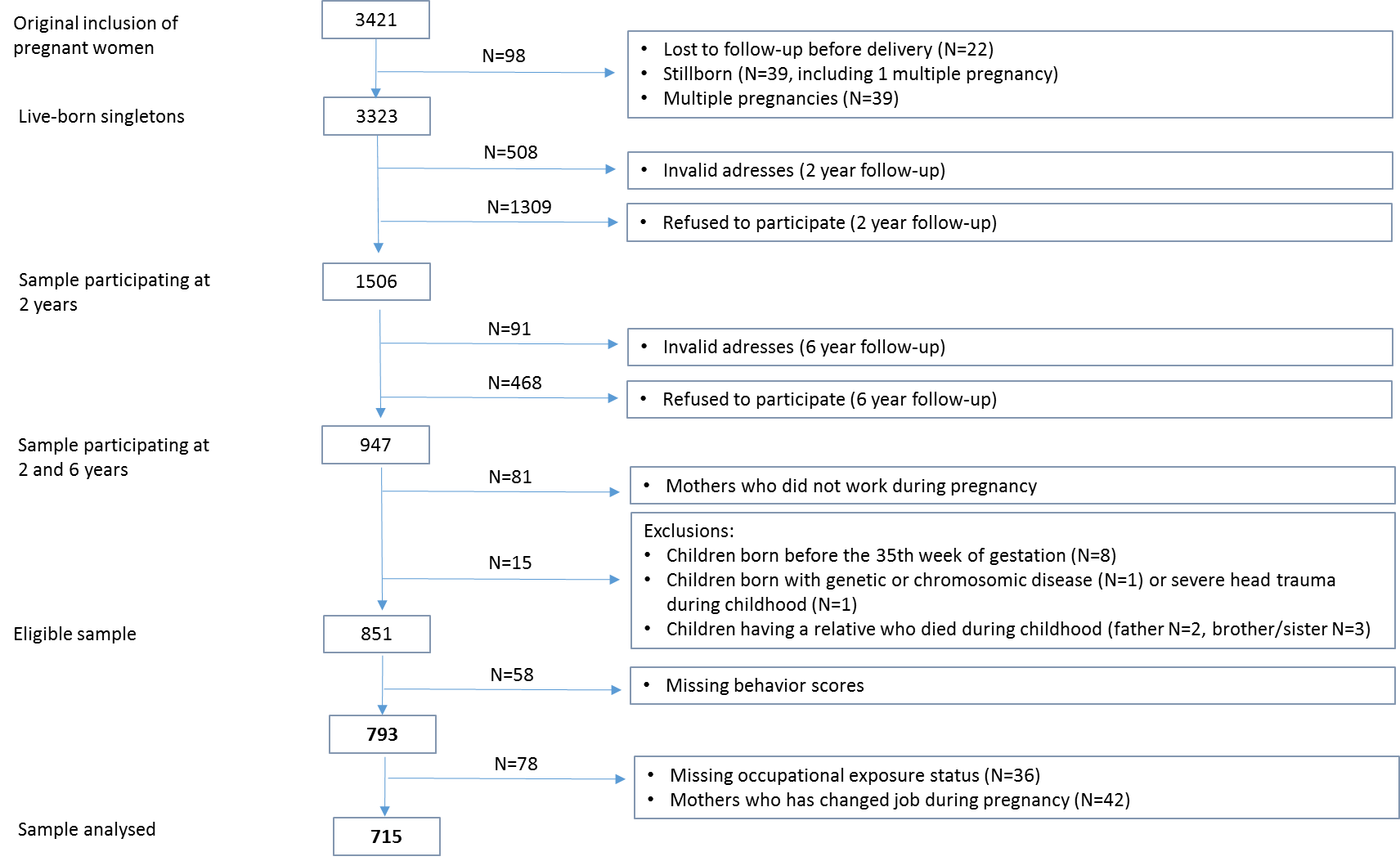
**

**Table S1.** Items used to assess the child behavior at age 2 in the PELAGIE cohort study, France, 2002-2013.

| **“During the last 3 months, would you says that your child”** | **Corresponding subscales** |
| --- | --- |
| Was easily distracted, had trouble sticking to any activity | Attention deficit /hyperactivity items |
| Couldn’t concentrate or hold his/her attention for a long period | (0 – 12 points) |
| Was inattentive |  |
| Couldn’t sit still, was restless or hyperactive |  |
| Fidgeted |  |
| Had difficulty awaiting for his/her turn in games |  |
| Got into fight | Aggression items |
| Physically attacked others | (0 – 6 points) |
| Hit, bit, kicked others |  |
| Was defiant or refused to comply with adults’ request or rules | Opposition items |
| Didn’t seem to feel guilty after misbehaving | (0 – 6 points) |
| Punishment didn’t change his/her behavior |  |
| Seemed to be unhappy or sad | Emotionality items |
| Was too fearful or anxious | (0 – 6 points) |
| Was worried |  |
| Helped someone who was injured | Prosocial items |
| Comforted a crying or an upset child (friend, brother or sister) | (0 – 6 points) |
| Helped another child (friend, brother or sister) who felt unwell |  |

*For more details, see* ^13^*. Score for each items were obtained from a 3-points Likert scale (0 = never, 1 = sometimes, 2 = often). Subscale scores were obtained by summing the score obtained for the corresponding items.*

**Table S2.** Items used to assess the mother-child interaction at age 2 in the PELAGIE cohort study, France, 2002-2013.

| Items | Score range |
| --- | --- |
| Do you sing with your child? | 1 - 5 |
| Do you read stories or show pictures in a book to your child? | 1 - 5 |
| Do you play with games at home with your child? | 1 - 5 |
| Do you take your child for walks? | 1 - 5 |
| Do you have physical games with your child? | 1 - 5 |

*Based on* ^13^*. Score for each items were obtained from a 5-points Likert scale (1 = never or almost never, 2 = less than once a week, 3 = once or twice a week, 4 = 3-5 times a week, 5 = every day or almost). Internal consistency was 0.75 for mother-child interaction. Unidimensionality was assessed with exploratory factorial analysis.*

**Table S3.** Groups of products related to occupational exposure to solvents (N= 715) in the PELAGIE Cohort, France, 2002-2013.

| Self-reported occupational tasks ^a^ | Total  N (%) | Group of occupational solvent exposure | |
| --- | --- | --- | --- |
|  |  | **occasional** | **regular** |
| Paints, lacquer | **706** |  |  |
| Occasional | 40 (5.7%) | 18 (12.9%) | 22 (10.0%) |
| Regular | 15 (2.1%) | 0 | 15 (6.8%) |
| Stripper | **700** |  |  |
| Occasional | 27 (3.9%) | 9 (6.5%) | 18 (8.2%) |
| Regular | 10 (1.4%) | 0 | 10 (4.6%) |
| Inks, dyes | **702** |  |  |
| Occasional | 43 (6.1%) | 24 (17.3%) | 19 (8.6%) |
| Regular | 28 (4.0%) | 0 | 28 (12.7%) |
| Glues, mastics, resins, adhesives | **703** |  |  |
| Occasional | 82 (11.7%) | 53 (38.1%) | 29 (13.2%) |
| Regular | 45 (6.4%) | 0 | 45 (20.5%) |
| Varnish (wood) | **702** |  |  |
| Occasional | 14(2.0%) | 4 (2.9%) | 10 (4.6%) |
| Regular | 1 (0.1%) | 0 | 1 (0.5%) |
| Wood processing products | **703** |  |  |
| Occasional | 13 (1.9%) | 6 (4.3%) | 7 (3.2%) |
| Regular | 3 (0.4%) | 0 | 3 (1.4%) |
| Cleaning products, detergents | **711** |  |  |
| Occasional | 109 (15.3%) | 87 (62.6%) | 22 (10.0%) |
| Regular | 152 (21.4%) | 0 | 152 (69.0%) |
| Diluent, grease remover | **694** |  |  |
| Occasional | 39 (5.6%) | 14 (10.1%) | 25 (11.4%) |
| Regular | 27 (3.9%) | 0 | 27 (12.3%) |
| Fuel, gasoline | **702** |  |  |
| Occasional | 21 (3.0%) | 9 (6.5%) | 12 (5.5%) |
| Regular | 14 (2.0%) | 0 | 15 (6.4%) |
| Textile treatment agents | **701** |  |  |
| Occasional | 5 (0.7%) | 2 (1.4%) | 3 (1.4%) |
| Regular | 5 (0.7%) | 0 | 5 (2.3%) |
| Cosmetics | **703** |  |  |
| Occasional | 21 (3.0%) | 3 (2.2%) | 18 (8.2%) |
| Regular | 13 (1.8%) | 0 | 13 (5.9%) |

^a^ Reading mode: column %, non-exposure is not presented.

Example: For the exposure to paints and, lacquers, 5.7% of the women declared occasional exposure and 2.1% declared regular exposure. This was the case for respectively 10.4% and 7.1% of the women classified as regularly exposed in our study.

| **Table S4.** Distribution of maternal occupations during pregnancy in the PELAGIE Cohort, France, 2002-2013. | | | | | | | | |
| --- | --- | --- | --- | --- | --- | --- | --- | --- |
|  | **All** | | **Group of occupational exposure to solvents during pregnancy** | | | | | |
| **Job titles** |  |  | No | | Occasional | | Regular | |
|  | N | % | N | % | N | % | N | % |
| Medical, dental, veterinarians, pharmacists | 48 | 6.8 | 26 | 7.4 | 10 | 7.3 | 12 | 5.5 |
| Nurses, midwives, medical x ray technicians | 64 | 9.0 | 9 | 2.6 | 11 | 8.0 | 44 | 20 |
| Nurses’ aides | 28 | 3.9 | 2 | 0.6 | 7 | 5.1 | 19 | 8.6 |
| Architects, engineers and related technicians | 24 | 3.4 | 18 | 5.1 | 5 | 3.6 | 1 | 0.5 |
| Statisticians, mathematicians, system analysts | 18 | 2.5 | 16 | 4.6 | 1 | 0.7 | 1 | 0.5 |
| Teachers | 97 | 13.7 | 42 | 11.9 | 30 | 21.7 | 25 | 11.4 |
| Social workers | 16 | 2.3 | 6 | 1.7 | 8 | 5.8 | 2 | 0.9 |
| Chemists, biologists and related workers | 29 | 4.1 | 8 | 2.3 | 3 | 2.2 | 18 | 8.2 |
| Jurists, journalists and related workers | 44 | 6.2 | 41 | 11.7 | 3 | 2.2 | 4 | 1.8 |
| Other professional and technical workers | 13 | 1.8 | 7 | 2.0 | 2 | 1.5 | 0 | 0.0 |
| Managerial workers | 8 | 1.1 | 6 | 1.7 | 2 | 1.5 | 0 | 0.0 |
| Clerical and related workers | 144 | 20.3 | 106 | 30.1 | 26 | 18.9 | 12 | 5.5 |
| Technical saleswomen, commercial travellers | 12 | 1.7 | 9 | 2.6 | 2 | 1.5 | 1 | 0.5 |
| Saleswomen, shop assistants | 42 | 5.9 | 17 | 4.8 | 10 | 7.3 | 15 | 6.8 |
| Other sales workers | 23 | 3.2 | 13 | 3.7 | 2 | 1.5 | 8 | 3.6 |
| Cleaners and helpers | 33 | 4.7 | 3 | 0.9 | 1 | 0.7 | 29 | 13.2 |
| Hairdressers, beauticians | 8 | 1.1 | 0 | 0.0 | 0 | 0.0 | 8 | 3.6 |
| Other service workers | 16 | 2.3 | 5 | 1.4 | 2 | 1.5 | 9 | 4.1 |
| Agricultural workers | 10 | 1.4 | 4 | 1.1 | 2 | 1.5 | 4 | 1.8 |
| Textile workers | 3 | 0.4 | 2 | 0.6 | 1 | 0.7 | 0 | 0.0 |
| Food and beverage processors | 3 | 0.4 | 2 | 0.6 | 1 | 0.7 | 0 | 0.0 |
| Electronic or metal processors | 9 | 1.3 | 4 | 1.1 | 2 | 1.5 | 3 | 1.4 |
| Material handlers and related equipment operators | 8 | 1.1 | 3 | 0.9 | 3 | 2.2 | 2 | 0.9 |
| Other production workers | 10 | 1.4 | 3 | 0.9 | 4 | 2.9 | 3 | 1.4 |
| Total ^a^ | 710 | 100 | 352 | 100 | 138 | 100 | 220 | 100 |

^a^ N=5 women with missing job title.

**Table S5.** Characteristics of samples across the PELAGIE Cohort follow-ups (France. 2002-2013).

|  |  | **Inclusion (N=3323)** | |  | **2 years (N=1506)** | |  | **6 years (N=947)** | |  | **Final sample (N=715)** | |
| --- | --- | --- | --- | --- | --- | --- | --- | --- | --- | --- | --- | --- |
| **Characteristics** |  | N | median [Q1-Q3] or % |  | N | median [Q1-Q3] or % |  | N | median [Q1-Q3] or % |  | N | median [Q1-Q3] or % |
| **Maternal age (years)** | - | 3323 | 29.9 [27- 32.9] |  | 1506 | 30.1 [27.3-33.1] |  | 947 | 30.6 [27.8-33.4] |  | 715 | 30.7 [28.0-33.4] |
| **Maternal education** | <12 years | 625 | 18.85 |  | 221 | 14.71 |  | 124 | 13.11 |  | 84 | 11.76 |
| **level** | 12 years | 623 | 18.79 |  | 278 | 18.51 |  | 162 | 17.12 |  | 114 | 15.97 |
|  | >12 years | 2067 | 62.35 |  | 1003 | 66.78 |  | 660 | 69.77 |  | 516 | 72.27 |
|  | Missing | 8 |  |  | 4 | |  | 1 | |  | 1 | |
| **Maternal prepregnancy BMI** | ≤25 kg/m² | 2747 | 82.67 |  | 1247 | 82.8 |  | 789 | 83.32 |  | 604 | 84.48 |
|  | >25 kg/m² | 576 | 17.33 |  | 259 | 17.2 |  | 158 | 16.68 |  | 111 | 15.52 |
| **Maternal tobacco consumption** | None | 2360 | 71.82 |  | 1129 | 75.72 |  | 732 | 77.96 |  | 547 | 77.04 |
| **(early pregnancy)** | <10 cig/day | 612 | 18.62 |  | 251 | 16.83 |  | 143 | 15.23 |  | 118 | 16.62 |
|  | ≥10 cig/day | 314 | 9.56 |  | 111 | 7.44 |  | 64 | 6.82 |  | 45 | 6.34 |
|  | Missing | 37 |  |  | 15 | |  | 8 | |  | 5 | |
| **Parity** | Nulliparous | 1473 | 44.49 |  | 646 | 43.12 |  | 386 | 40.8 |  | 299 | 41.82 |
|  | Parous | 1838 | 55.51 |  | 852 | 56.88 |  | 560 | 59.2 |  | 416 | 58.18 |
|  | Missing | 12 |  |  | 8 | |  | 1 | |  |  |  |
| **Child’s sex** | Boys | 1669 | 50.24 |  | 767 | 50.96 |  | 484 | 51.16 |  | 378 | 52.87 |
|  | Girls | 1654 | 49.76 |  | 739 | 49.04 |  | 463 | 48.84 |  | 337 | 47.13 |
| **Birth weight** | - | 3321 | 3385 [3110-3700] |  | 1504 | 3400 [3120-3720] |  | 945 | 3410 [3120-3730] |  | 714 | 3420 [3150-3730] |
| **Breastfeeding** | None |  | - |  | 515 | 34.82 |  | 306 | 32.31 |  | 232 | 32.45 |
|  | <6 weeks |  | - |  | 643 | 43.48 |  | 422 | 44.56 |  | 331 | 46.29 |
|  | ≥6 weeks |  | - |  | 321 | 21.70 |  | 219 | 23.13 |  | 152 | 21.26 |
|  | Missing |  | - |  | 27 | |  |  | |  |  | |
| **Birth weight** | - | 3321 | 3385 [3110-3700] |  | 1504 | 3400 [3120-3720] |  | 945 | 3410 [3120-3730] |  | 714 | 3420 [3150-3730] |
| **Solvent prenatal exposure** | None | 1385 | 50.04 |  | 640 | 49.31 |  | 415 | 50.12 |  | 356 | 49.79 |
|  | Occasional | 552 | 19.94 |  | 263 | 20.26 |  | 159 | 19.2 |  | 139 | 19.44 |
|  | Regular | 831 | 30.02 |  | 395 | 30.43 |  | 254 | 30.68 |  | 220 | 30.77 |
|  | Missing | 555 |  |  | 208 |  |  | 119 |  |  |  |  |
| **Behavior subscales at 2 years** |  |  |  |  |  |  |  |  |  |  |  |  |
| **Emotional symptoms** | - | - | - |  | 1484 | 4 [3-5] |  | 934 | 4 [3-5] |  | 715 | 4 [3-5] |
| **Attention deficit/Hyperactivity** | - | - | - |  | 1442 | 11 [9-13] |  | 902 | 11 [9-13] |  | 715 | 11 [9-13] |
| **Aggression** | - | - | - |  | 1471 | 5 [3-6] |  | 922 | 4 [3-6] |  | 715 | 4 [3-6] |
| **Opposition** | - | - | - |  | 1467 | 6 [5-7] |  | 924 | 6 [5-7] |  | 715 | 6 [5-7] |

**Table S6**. Associations between behavior latent traits at 2 and 6 years (crude and adjusted models). PELAGIE Cohort. France. 2002-2013. N=715.

|  | Latent traits at age 6 | | | | | | | |
| --- | --- | --- | --- | --- | --- | --- | --- | --- |
|  | **Internalizing behavior** | | | | **Externalizing behavior** | | | |
| Latent traits at age 2 | | Estimate^a^ | | 95% CI | | Estimate^a^ | | 95% CI |
| Internalizing behavior | | | | | | | | |
| Crude model^b^ | | 0.31 | 0.19, 0.42 | | | -0.04 | -0.13, 0.06 | |
| Adjusted model^c^ | | 0.27 | 0.15, 0.38 | | | -0.07 | -0.16, 0.02 | |
| Externalizing behavior | | | | | | | | |
| Crude model^b^ | | 0.21 | 0.07, 0.34 | | | 0.66 | 0.56, 0.77 | |
| Adjusted model^c^ | | 0.20 | 0.06, 0.33 | | | 0.62 | 0.51, 0.73 | |

Abbreviations: 95% CI, 95% confidence interval

^a^ SEM standardized regression coefficients and 95% confidence intervals.

**^b^** Crude model: unadjusted structural equation modeling of behavior at age 2 and 6; Fit indices: Chi Square=34.808, df=15, p=0.003; RMSEA=0.043; GFI=0.998; CFI=0.978; SRMR=0.034.

**^c^**Adjusted model: SEM regression model adjusted for sex, education level, maternal age, breastfeeding duration, smoking during pregnancy, parity, and mother-child interaction score, and including the prenatal self-reported exposure to solvents; Fit indices: Chi Square=117.894, df=63, p=0.000; RMSEA=0.035; GFI=0.999; CFI=0.953; SRMR=0.027

**Table S7**. Structural Equation Modeling of behavior at ages 2 and 6 in the PELAGIE Cohort. France. 2002-2013 – Factor loadings for the latent traits at ages 2 and 6 (crude and adjusted model. n=715)

| **Latent variables** | **Observed variables** |  | **Factor loadings** | | | | | |
| --- | --- | --- | --- | --- | --- | --- | --- | --- |
|  |  | **Model^a^** | Estimate | | 95% CI | Stand. Est. | 95% CI | % of variance explained by latent trait |
| **AGE 2** |  |  |  | | |  | |  |
| Internalizing behavior | Emotionality score | Crude | 1 |  | | 1 |  | 1 |
|  |  | Adjusted | 1 |  | | 1 |  | 1 |
| Externalizing behavior | Attention deficit/hyperactivity score | Crude | 1 |  | | 0.70 | 0.63, 0.78 | 0.50 |
|  |  | Adjusted | 1 |  | | 0.71 | 0.64, 0.78 | 0.50 |
|  | Aggression score | Crude | 0.36 | 0.27, 0.45 | | 0.44 | 0.36, 0.53 | 0.16 |
|  |  | Adjusted | 0.38 | 0.29, 0.47 | | 0.45 | 0.37, 0.53 | 0.20 |
|  | Opposition score | Crude | 0.47 | 0.39, 0.56 | | 0.63 | 0.57, 0.70 | 0.40 |
|  |  | Adjusted | 0.47 | 0.39, 0.56 | | 0.61 | 0.55, 0.68 | 0.37 |
| **AGE 6** |  |  |  | | |  | |  |
| Internalizing | Emotional symptoms score | Crude | 1 |  | | 0.63 | 0.51, 0.74 | 0.35 |
|  |  | Adjusted | 1 |  | | 0.64 | 0.53, 0.75 | 0.41 |
|  | Peer-relationship problems score | Crude | 0.62 | 0.41, 0.83 | | 0.52 | 0.42, 0.62 | 0.27 |
|  |  | Adjusted | 0.65 | 0.46, 0.84 | | 0.51 | 0.42, 0.60 | 0.26 |
| Externalizing | Hyperactivity-inattention score | Crude | 1 |  | | 0.61 | 0.53, 0.69 | 0.38 |
|  |  | Adjusted | 1 |  | | 0.64 | 0.56, 0.71 | 0.41 |
|  | Conduct problems score | Crude | 0.77 | 0.62, 0.92 | | 0.71 | 0.63, 0.78 | 0.49 |
|  |  | Adjusted | 0.74 | 0.61, 0.87 | | 0.68 | 0.61, 0.75 | 0.46 |

Abbreviation: 95% CI, 95% confidence interval; Stand. Est., SEM standardized estimates of the factor loadings.

**^a^** Crude model: unadjusted structural equation modeling of behavior at age 2 and 6; Adjusted model: SEM regression models adjusted for sex, education level, maternal age, breastfeeding duration, smoking during pregnancy, parity, and mother-child interaction score, and including the prenatal self-reported exposure to solvents.

**Table S8**. Structural Equation Modeling of behavior at ages 2 and 6 in the PELAGIE Cohort. France. 2002-2013 – Factor loadings for the latent traits at ages 2 and 6 (crude model) in boys (N=379) and girls (N=336).

| **Latent variables** | **Observed variables** | **Standardized factor loadings ^a^** | | | |
| --- | --- | --- | --- | --- | --- |
|  |  | **In Boys**  Estimate | 95% CI | **In Girls**  Estimate | 95% CI |
| **AGE 2** |  |  | |  | |
| Internalizing behavior | Emotionality score | 1 |  | 1 |  |
| Externalizing behavior | Attention deficit/hyperactivity score | 0.72 | 0.61, 0.82 | 0.68 | 0.58, 0.79 |
|  | Aggression score | 0.40 | 0.29, 0.52 | 0.46 | 0.34, 0.59 |
|  | Opposition score | 0.63 | 0.54, 0.71 | 0.65 | 0.55, 0.76 |
| **AGE 6** |  |  | |  | |
| Internalizing behavior | Emotional symptoms score | 0.57 | 0.46, 0.69 | 0.62 | 0.51, 0.74 |
|  | Peer-relationship problems score | 0.78 | 0.66, 0.89 | 0.60 | 0.49, 0.7 |
| Externalizing behavior | Hyperactivity-inattention score | 0.58 | 0.44, 0.72 | 0.76 | 0.54, 0.99 |
|  | Conduct problems score | 0.59 | 0.45, 0.73 | 0.41 | 0.27, 0.55 |

^a^ SEM standardized regression coefficients and 95% confidence intervals. Crude model: unadjusted structural equation modeling of behavior at age 2 and 6; Model fit indices: In boys (n=379): Chi Square=33.891, df=15, p=0.004; RMSEA=0.058; GFI=0.996; CFI=0.964; SRMR=0.046; In girls (n=336): Chi Square=9.181, df=15, p=0.868; RMSEA=0.000; GFI=0.999; CFI=1.000; SRMR=0.026

**Table S9**. Associations between behavior latent traits at age 6 and age 2 in boys (N=379) and girls (N=336) (crude model), PELAGIE Cohort, France, 2002-2013.

|  |  | Latent traits at age 6 | | | |
| --- | --- | --- | --- | --- | --- |
|  |  | Internalizing behavior | | Externalizing behavior | |
| Latent traits at age 2 |  | Estimate^a^ | 95% CI | Estimate^a^ | 95% CI |
| Internalizing behavior | Boys | 0.26 | 0.10, 0.41 | 0.01 | -0.11, 0.14 |
|  | Girls | 0.33 | 0.18, 0.49 | -0.09 | -0.22, 0.05 |
| Externalizing | Boys | 0.26 | 0.08, 0.44 | 0.61 | 0.47, 0.75 |
| behavior | Girls | 0.14 | -0.04, 0.32 | 0.73 | 0.58, 0.89 |

^a^ SEM standardized regression coefficients and 95% confidence intervals. Crude model: unadjusted structural equation modeling of behavior at age 2 and 6; Model fit indices: In boys (n=379): Chi Square=33.891, df=15, p=0.004; RMSEA=0.058; GFI=0.996; CFI=0.964; SRMR=0.046; In girls (n=336): Chi Square=9.181. df=15. p=0.868; RMSEA=0.000; GFI=0.999; CFI=1.000; SRMR=0.026

**Table S10**. Sex-Stratified Total Associations Between Occupational Solvent Exposure During Pregnancy and Child Behavior at Age 2 and Age 6 (N=379 Boys and N=336 Girls, PELAGIE cohort, France, 2002-2013).

|  | |  | | In boys | | | | | | | | | | In girls | | | | | | | | |
| --- | --- | --- | --- | --- | --- | --- | --- | --- | --- | --- | --- | --- | --- | --- | --- | --- | --- | --- | --- | --- | --- | --- |
| Behavior traits | **Prenatal Exposure** | |  | | **Association**  **at age 2** | | | | **Total association**  **at age 6** | | | |  | | **Association**  **at age 2** | | | | **Total association**  **at age 6** | | | |
|  |  |  | N | | Stand. Coeff.^a^ | | | 95% CI | Stand. Coeff.^a^ | | | 95% CI | N | | Stand. Coeff.^a^ | | | 95% CI | Stand. Coeff.^a^ | | | 95% CI |
| Internalizing | **None** | | 198 | | ref | |  | | ref | |  | | 158 | | ref | |  | | ref | |  | |
| behavior | **Occasional** | | 63 | | 0.05 | | -0.22. 0.32 | | 0.21 | | -0.15. 0.57 | | 76 | | 0.20 | | -0.04. 0.44 | | 0.19 | | -0.17. 0.55 | |
|  | **Regular** | | 118 | | 0.14 | | -0.10. 0.37 | | 0.13 | | -0.22. 0.44 | | 102 | | -0.07 | | -0.33. 0.19 | | 0.01 | | -0.32. 0.35 | |
| Externalizing | **None** | | 198 | | ref | |  | | ref | |  | | 158 | | ref | |  | | ref | |  | |
| behavior | **Occasional** | | 63 | | 0.23 | | -0.09. 0.55 | | 0.15 | | -0.21. 0.51 | | 76 | | 0.44 | | 0.11. 0.77 | | 0.32 | | -0.04. 0.68 | |
|  | **Regular** | | 118 | | 0.23 | | -0.05. 0.51 | | -0.06 | | -0.34. 0.22 | | 102 | | 0.26 | | -0.07. 0.59 | | 0.31 | | -0.05. 0.66 | |
|  | |  | |  | |  | | | |  | | | |  | |  | | | |  | | |

Abbreviations: 95% CI. 95% Confidence Interval; Stand. Coeff.. SEM-based adjusted

^a^ SEM-based adjusted standardized regression coefficients. All SEM regressions were adjusted for education level. maternal age. breastfeeding duration. smoking during pregnancy. parity. and mother-child interaction score. Fit indices: In boys. Chi Square=104.861. df=59. P<0.01; RMSEA=0.045; GFI=0.999; CFI=0.932; SRMR=0.037; In girls. Chi Square=53.870. df=59. P=0.665; RMSEA=0.000; GFI=0.999; CFI=1; SRMR=0.028

**Table S11**. Comparison of children participating at the 2 year and 6 year follow-ups with those participating only at the 2 year follow-up (PELAGIE Cohort. France. 2002-2013).

|  |  | **Children participating only at age2** |  | **Children participating at age 2 and age 6** | p-value |
| --- | --- | --- | --- | --- | --- |
|  |  | N=445 ^e^ |  | N=771 ^e^ |  |
| **Behavior subscales** ^a^ |  |  |  |  |  |
| Emotion |  | 4.4 (1.2) |  | 4.5 (1.2) | 0.42 ^b^ |
| Hyperactivity/Inattention |  | 10.9 (2.5) |  | 10.9 (2.6) | 0.10 ^c^ |
| Aggression |  | 4.6 (1.5) |  | 4.6 (1.4) | 0.39 ^b^ |
| Opposition |  | 5.7 (1.3) |  | 5.7 (1.3) | 0.94 ^b^ |
|  |  |  |  |  |  |
| **Solvent prenatal exposure** | |  |  |  | 0.47 ^d^ |
| None |  | 211 (47%) |  | 382 (50%) |  |
| Occasional |  | 99 (22%) |  | 149 (19%) |  |
| Regular |  | 135 (30%) |  | 240 (31%) |  |

^a^ Behavior subscales from the CBCL (Child Behavior Checklist) and PSBQ (Preschool Social Behavior Questionnaire)

^b^ Wilcoxon test; ^c^ Student test; ^d^ Chi-square test

^e^ Children with complete behavior and exposure data (445 out of 559 participants at 2 years only and 771 out of 947 participating at 2 and 6 years)
